# Comparative analysis of radiation therapy and surgical approaches for resectable cancers: a narrative review

**DOI:** 10.1097/MS9.0000000000004948

**Published:** 2026-04-23

**Authors:** Akshit Bhambri, Ozoemena Z. Akah, Kirtan Soni, Fnu Anirudh, Shubh Mehta, Hend Makky, Divyanshi Vijay Kumar, Rubén Darío Páramo Cardona, Francisco Zarra, Arlette Villalobos, Parvinder Kaur

**Affiliations:** a Medical College & Hospital, Hyderabad, Telangana, India; b HCA Mountain View, Hyderabad, Telangana, India; c Internal Medicine/Emergency Medicine, GMERS Medical College, Hyderabad, Telangana, India; d All India Institute of Medical Sciences, Hyderabad, Telangana, India; e Department of Internal Medicine, B.J. Medical College and Civil Hospital, Hyderabad, Telangana, India; f Faculty of Medicine, Assiut University, Hyderabad, Telangana, India; g Smt. Nathida Hargovandas Lakhmichand Municipal Medical College, Hyderabad, Telangana, India; h Department of Hand Surgery, Orthopedic Surgery, Hospital Alemán, Argentina; i University of Buenos Aires, Buenos Aires, Argentina; j Ponce Health Sciences University School of Medicine, Hyderabad, Telangana, India; k Crimean State Medical University named after Sal. Georgievsky, Simferopol, Ukraine

**Keywords:** patient outcomes, precision oncology, radiation therapy, resectable cancers, surgical resection, treatment efficacy

## Abstract

This narrative review compares radiation therapy and surgical approaches for resectable cancers, focusing on their efficacy, safety, and patient outcomes. Surgical resection remains the gold standard for many localized cancers, offering the potential for complete tumor removal and long-term survival. However, radiation therapy has emerged as a valuable adjunct or alternative, especially for tumors that are difficult to access surgically or in patients with comorbidities that limit surgical candidacy. The review highlights key factors such as tumor type, location, and patient performance status when selecting the optimal treatment approach. It also discusses recent advances in both modalities, including precision surgery and image-guided radiation therapy, which have improved the therapeutic index. Although both strategies can offer curative potential, a multidisciplinary approach tailored to individual patient needs is critical for achieving the best outcomes.

**Graphical abstract available at:** http://links.lww.com/MS9/B119

## Graphical Abstract

**Figure:**
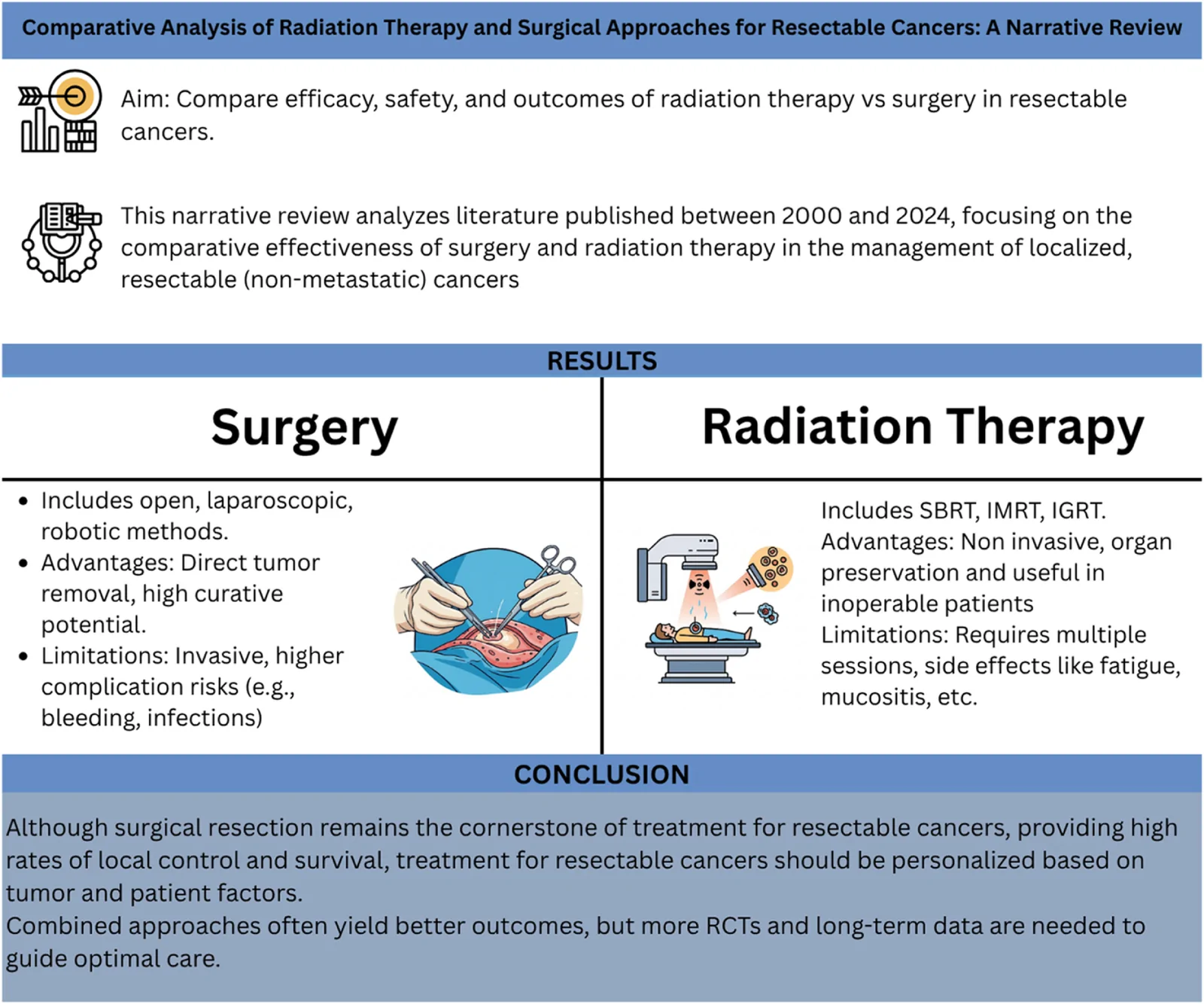

## Introduction

Cancer remains one of the leading causes of morbidity and mortality worldwide, posing a formidable challenge to modern healthcare. In recent decades, advances in diagnostic methods and therapeutic strategies have revolutionized the management of various malignancies, particularly resectable cancers^[^1,2^]^. Resectable cancers are those in which the tumor is localized and confined to a specific anatomical region, allowing for potential surgical removal with curative intent or to significantly prolong survival. The choice of treatment is a nuanced decision, influenced by tumor characteristics, patient preferences, and a multidisciplinary team approach. Among the most common resectable cancers are breast, colorectal, and prostate cancers, where screening protocols are well-established, leading to higher rates of early detection and resectability. In contrast, cancers like lung and pancreatic cancers, which lack routine screening measures, often present at more advanced stages, limiting the feasibility of surgical intervention^[^3^]^. The purpose of this review is to evaluate the importance of radiotherapy (RT) and resection for the treatment of cancer.

HIGHLIGHT

- SBRT achieves ~90% 1-year local control in early-stage resectable non-small cell lung cancer.
- AI-driven radiation planning cuts planning time by ~60% while improving quality.
- Robot-assisted surgery reduces positive margin rates in prostate cancer.
- Fluorescence-guided surgery improves complete tumor resection up to 95%.

## Review

This narrative review compares the efficacy, safety, and outcomes of radiation therapy and surgical approaches for resectable cancers. A literature search was conducted across PubMed, Scopus, Web of Science, and Embase from January 2010 to August 2025, using MeSH terms and keywords such as “Radiation Therapy,” “Surgical Resection,” “Resectable Cancers,” and “Patient Outcomes.” Boolean operators (AND, OR) refined results to focus on studies comparing these modalities in resectable malignancies. Only peer-reviewed, English-language articles were included, excluding studies on non-resectable cancers or non-oncologic conditions. Two reviewers independently screened studies, with a third resolving conflicts. Quality was assessed using the Cochrane Risk of Bias Tool for randomized controlled trials (RCTs), the Newcastle-Ottawa Scale for observational studies, and the GRADE approach. Author A.B led search strategy and extraction, Author O.Z.A refined MeSH terms, and Author K.S oversaw quality assessment. This methodology ensures a systematic synthesis of evidence on radiation therapy and surgery for resectable cancers.

## Treatment modalities: radiation therapy and surgery

### Radiation therapy

Radiation therapy, a cornerstone of non-surgical cancer management, leverages high-energy ionizing radiation to induce DNA damage and cell death in malignant cells, decreasing the risk of spreading to other adjacent tissues. Techniques such as stereotactic body radiation therapy (SBRT) and intensity-modulated radiation therapy (IMRT) have significantly enhanced precision, allowing for targeted tumor irradiation while sparing adjacent healthy tissues^[^4^]^. Radiation therapy is administered either by external beam radiation therapy or brachytherapy, which is given internally^[^5^]^. Nevertheless, radiation therapy is not devoid of adverse effects, which may range from acute reactions like dermatitis and fatigue to long-term sequelae including fibrosis and damage to adjacent organs^[^6^]^. Moreover, radiation therapy may be less effective as a monotherapy, particularly for large or aggressive tumors, prompting consideration of multimodal treatment strategies.

### Surgical approaches

Surgical resection has traditionally been the fundamental of curative cancer therapy, particularly for solid tumors. Advances in surgical techniques, including minimally invasive methods such as laparoscopic and robotic-assisted surgery, have reduced perioperative morbidity and mortality, shortened recovery times, and improved postoperative outcomes^[^7^]^. Despite these advancements, surgery carries inherent risks, including bleeding, infection, and long-term complications, particularly when performed on anatomically challenging sites like the liver, lungs, or pancreas^[^8^]^. Furthermore, the potential for incomplete resection and recurrence necessitates the integration of adjuvant therapies to maximize patient outcomes.

Different surgical strategies can be implemented. The conventional approach is the open surgery method. However, science and technology innovative methods such as laparoscopic or robotic-assisted techniques can be used. These methods are not only less invasive (with minimal incision size) but also shorten the recovery time and speed up the healing process. Moreover, it has fewer complications post-surgery though the technique has proven to be effective in cancer management with better patient outcomes^[^9,10^]^. Furthermore, medical procedures are not very safe as they may lead to infection, bleeding, and chronic complications, particularly when being done close to the lungs, liver, or pancreas^[^8^]^.

Comprehensive care including surgery, radiation, and medication therapies, tailored for the specific needs of the patients, is prescribed to have an effective treatment plan for better patient outcomes thus, reducing risk to minimal^[^7,11^]^. Given the dynamic nature of cancer treatment and the emergence of novel therapeutic modalities, a comprehensive evaluation of the relative merits and limitations of radiation therapy and surgery for resectable cancers is essential. This narrative review aims to synthesize current evidence, compare outcomes between these treatment modalities, and explore future directions in optimizing cancer management. By critically analyzing recent advances and clinical outcomes, this review seeks to inform evidence-based decision-making and highlight areas for future research in the treatment of resectable cancers.

While surgery remains the most established treatment approach, advances in radiation therapy – such as SBRT and IMRT – have significantly improved patient outcomes by enabling more precise tumor targeting and minimizing damage to surrounding healthy tissues^[^12^]^.

### Indications for radiation therapy in resectable cancers

RT is performed by administering nuclear radiation to abnormal cancer cells, thereby inhibiting their growth and ability to multiply, ultimately leading to cell death. This approach serves both curative and palliative purposes and is often combined with surgery and chemotherapy^[^13,14^]^. Modern RT modalities, including Image-Guided Radiation Therapy, Adaptive Radiation Therapy, and Volumetric Modulated Arc Therapy, have enhanced treatment precision and improved outcomes^[^15^]^.

RT with curative intent is typically applied when tumors are localized and surgically resectable. It is a standard option in early-stage non-small cell lung cancer (NSCLC) for patients who are medically ineligible for surgery or have advanced disease^[^16,17^]^. Outcomes are further improved when RT is integrated with surgery and systemic therapies^[^18,19^]^.

In neoadjuvant and adjuvant settings, RT is administered before surgery to shrink tumor size and facilitate resection or after surgery to eliminate residual disease and reduce recurrence risk. The German Rectal Cancer Study highlighted the advantages of preoperative chemoradiotherapy, showing reduced local recurrence and fewer postoperative complications^[^16^]^. Such strategies are particularly useful in locally advanced cancers where minimizing tumor burden improves operability.

RT is also tailored to specific cancer types and stages. In breast cancer, it is routinely prescribed following lumpectomy or mastectomy. For high-risk head and neck cancers, postoperative RT (PORT) combined with chemotherapy provides significant benefit^[^20^]^. Similarly, in anal and laryngeal cancers, organ-preserving RT achieves excellent outcomes^[^21^]^, while in prostate cancer it contributes to improved survival^[^22^]^. However, treatment often requires daily sessions over several weeks, affecting compliance and well-being. Common side effects include fatigue and skin reactions^[^23^]^.

### Indications for surgical approaches in resectable cancers

Surgical treatment with curative intent – also termed as radical treatment – aims to achieve complete tumor eradication, with anticipated survival benefits assessed through models such as Kaplan–Meier curves^[^24^]^. Patient comorbidities and overall health must be factored into candidacy^[^25^]^. Examples include radical prostatectomy, excision of early-stage non-melanocytic skin cancers^[^26^]^, resection of thymic malignancies^[^27^]^, and combined trimodal therapy for esophageal or gastroesophageal cancers^[^25^]^.

Surgery is often the first-line treatment for resectable tumors in accessible locations such as the skin, breast, prostate, and cervix. Non-melanocytic skin cancers are highly prevalent, with millions of cases diagnosed annually in the US alone, and early surgical excision achieves near 99% success^[^26,28^]^. Similarly, breast-conserving surgery or mastectomy is curative in early-stage disease^[^29,30^]^. Prostatectomy and hysterectomy remain standard approaches for prostate and cervical cancers, respectively^[^25,31^]^.

The main benefits of surgery include its high cure rates in localized solid tumors and its ability to provide definitive management. Nonetheless, it carries risks related to tumor location, size, and proximity to vital structures, making it less suitable for advanced disease or tumors in critical regions such as brain gliomas^[^32^]^. Advances such as breast-conserving surgery combined with systemic therapies^[^29^]^ and 3D navigation-guided resections for liver and spine tumors^[^33,34^]^ have improved safety and outcomes.

Candidacy for surgery depends on patient characteristics, tumor biology, and the availability of supportive therapies. General condition, nutrition, and comorbidities affect surgical tolerance, while macroscopic and microscopic tumor features – including location, margins, vascularity, and aggressiveness – inform feasibility. Surgery may be pursued with curative or palliative intent. Even when complete resection is not possible, combining surgery with RT, chemotherapy, or hormonal therapy can yield survival benefits similar to radical surgery, as demonstrated in select cases such as HER2^+^ breast cancer and hepatocellular carcinoma^[^35,36^]^.

## Comparative outcomes

### Efficacy

**RT** facilitates downstaging, reduces metastasis, and improves locoregional control, making it effective in aggressive and locally advanced tumors. For example, in resectable stage IIIA NSCLC, PORT demonstrated a survival benefit in patients with N2 disease, >5 positive lymph nodes, visceral pleural invasion, or tumors >3 cm in size, whereas patients without N2 involvement derived little benefit^[^37,38^]^. Similarly, in gastroesophageal junction (GEJ) cancers, preoperative RT or chemoradiotherapy enhanced tumor regression compared with surgery alone, which is often associated with higher recurrence.

Surgery, being the cornerstone for potentially curative treatment, is limited by tumor resectability and patient suitability. In pancreatic ductal adenocarcinoma, only 20% of patients are resectable, with a 5-year survival rate of ~20%. Elderly patients (>70 years) undergoing pancreaticoduodenectomy experience higher recurrence and complications, though surgery remains justified in younger patients^[^39^]^. Conversely, chemoradiotherapy has demonstrated CA19-9 reduction, suggesting curative potential^[^40^]^. In NSCLC, lobectomy remains the gold standard for stage IA disease, achieving a 73% 5-year survival, whereas sublobar resection is reserved for selected cases^[^41^]^.

RT expands eligibility for curative treatment by downstaging, while surgery provides definitive local control when resection is feasible. Both modalities complement each other in multimodal protocols.

### Overall and disease-free survival

RT has shown favorable survival outcomes. In early oropharyngeal cancer, 3- and 5-year overall survival (OS) rates with RT were 77 and 71%, respectively, with locoregional-free survival at 71–75%^[^42^]^. In gastric cancer, perioperative RT improved 1- and 3-year survival (83–84% and 53–64%) compared with no RT (66% and 45%)^[^43^]^.

Surgery also achieves durable survival outcomes. In early-stage squamous cell carcinoma (cT1/T2 and cN1/0), surgical patients had 3- and 5-year OS rates of 71 and 59%, with locoregional-free survival of 66 and 50% respectively. Median OS and DFS were 6.5 and 6.1 years^[^42^]^.

Although surgery generally yields durable long-term control in resectable tumors, RT improves outcomes in patients with high recurrence risk or where surgery is not feasible. Combining the two enhances both OS and DFS in several tumor types.

### RT-related complications

RT complications are dose- and site-dependent. Acute effects [skin erythema, mucositis, and gastrointestinal (GI) irritation] resolve in weeks, whereas late effects (fibrosis, necrosis, vascular damage, xerostomia, pneumonitis, myocardial fibrosis, and rectal bleeding) may persist^[^44^]^. Secondary malignancies occur in two peaks: early hematological cancers and later solid tumors.

### Surgery-related complications

Surgical morbidity varies by site and patient profile. In rectal cancer, anastomotic leakage and wound issues are common, whereas abdominal surgeries carry risks of adhesions, SBO, infections, hemorrhage, thrombosis, and ileus. Systemic complications include respiratory dysfunction, urinary issues, and neurological changes, especially in elderly patients^[^45,46^]^.

RT tends to cause chronic but manageable toxicities, whereas surgery carries higher perioperative morbidity and systemic risks. RT late effects may emerge years later, while surgical complications typically appear early in the postoperative course.

### Side effects and recovery times

In radiation therapy, the quality-of-life (QOL) posttreatment is influenced by the dose administered. The minimum tolerated dose (TD5/5) and maximum tolerated dose (TD50/5) refer to the doses at which life-altering complications occur in 5 and 50% of patients, respectively. Factors such as age, anemia, immunity, BMI, infection, and comorbidities affect the development of late symptoms^[^44^]^.

In a 175-patients retrospective study for resectable NSCLC, propensity score matching was done commonly after neoadjuvant chemo immunotherapy, and a comparison of RT and surgical resection was done. In the comparison, it was found that level 3–4 treatment related adverse effects occurred in 62.5% of RT patients and 55% of surgical patients, in which treatment related pneumonitis/pneumonia was seen significantly in 7.5% of the RT group and 2.5% of the surgery group, respectively; level 5 TRAE were not noted^[^47^]^. In another case study of early oropharyngeal cancer, out of 61 patients, 37 had received adjuvant therapy with a further division of 24 receiving primary RT and 13 chemoradiotherapy (CRT). Modalities like intensity modulated RT or 3D conformal RT were chosen, and around 19 patients (34%) were treated with primary RT showed acute grade-3 toxic side effects, such as mucositis, dermatitis, xerostomia, dysphagia, etc. They were seen in patients who had a common primary or adjuvant setting of RT (32 and 37%). In contrast, higher numbers of toxic side effects were seen in patients who received CRT (56%) compared to those who received primary RT (25%). In severe late toxic side effects, grade 4 osteoradionecrosis of the jaw was reported out six patients, three patients had received primary RT, and three received adjuvant RT. It was noted that CRT-treated patients had higher chances of osteoradionecrosis than IMRT-treated patients. This tells us that the choice of RT also matters when it comes to side effects and not just the setting or modality; in this case, surgery had not shown any treatment-related adverse effects^[^42^]^.

### RT-reported outcomes

In a prospective cohort study, patients with borderline resectable or locally advanced pancreatic cancer undergoing RT were taken. According to the MD Anderson Symptom Inventory for GI cancer, fatigue, mouth dryness, numbness, difference in taste, and decreased appetite were the five most severe symptoms on the panel, but pre- and postoperative radiation did not show any significant differences in patient-reported outcomes^[^48^]^. Similarly in a study by Achim *et al*, patients who received unimodality, either transoral robotic surgery (TORS) or CRT alone or PORT alone, showed better results in the QOL. CRT and PORT show better patient-reported outcomes in head and neck cancers as they provided better locoregional coverage and OS; whereas in long-term patient-reported outcomes, mucosal and soft tissue damages were also reported^[^49^]^.

### Surgical reported outcomes

In a study of head and neck cancer where the postsurgery QOL impacts were checked through five questionnaires, it was determined that a longer length of surgery or hospital stay led to higher depression scores. Around 32 and 20% of respondents showed abnormal scores of depression and anxiety, respectively; body concerning issues were also common, especially in those candidates who required reconstruction^[^50^]^. Similarly, in a study of resectable esophageal cancer, patients who underwent open esophagectomy showed higher risks of major pulmonary and other complications compared to those who had multimodal procedures with higher values of pain and fatigue. Higher values of major complications, pain, and insomnia were also reported in 1-year OS with *P*-values of 0.035 and 0.031, respectively; esophageal adenocarcinoma shows decreased pulmonary complications compared to esophageal squamous cell carcinomas^[^51^]^. As a result, pain, fatigue, insomnia, and squamous cell pathology are considered as poor prognostic factors of surgery. A phase-2 randomized trial for early stage squamous cell carcinoma of the oropharynx has shown a positive association of dysphagia based QOL and surgical resection. This was measured through MD Anderson dysphagia inventory unimodality showing better patient-reported outcomes^[^52^]^ (Table 1).

**Table 1:** Comparative overview of recent innovations in radiotherapy, surgery, and combined approaches, highlighting key advantages, challenges, and future potential.

Treatment modality	Key innovation	Advantages	Challenges	Future potential
Radiation therapy	Stereotactic body radiation therapy	Accuracy Reduced damage to surrounding tissue 90% local control rate at 1 year for early-stage NSCLC^[^53^]^	Restricted to specific cancers and stages Needs specific apparatus	Widening scope to other cancers Integration with immunotherapy
	AI-driven planning	60% decrease in planning time Upgraded plan quality^[^54^]^	Requirement for comprehensive accurate data sets Combination with current systems	Customized treatment strategies Real time strategy adaptation
Surgery	Robot-assisted surgery	Improved accuracy and visual interpretation Reduced positive surgical margin rates in prostatectomy^[^55^]^	Large startup expenses -Competency curve for surgeons	Uses in multiple cancers Incorporation of AI for decision-making
	Fluorescence-guided surgery	Better visualization of tumor margin Enhanced complete resection rates (95% vs 80%)^[^56^]^	Restricted to particular tumor types Requirement for particular imaging agents	Advancement of new fluorescent markers Integration with superior imaging techniques
Combined Approaches	Neoadjuvant radiotherapy + surgery	Lower local recurrence rates in rectal cancer^[^57^]^ Possibility of organ conservation	Ideal timing and arrangement of therapies Handling treatment-induced toxicities	Tailored-integrated treatment plans Incorporation of immunotherapy and targeted treatments

### Case studies and clinical evidence

Surgery is the primary curative method for solid tumors such as pancreatic, esophageal, and cervical cancers, with total resection providing the best chance for long-term survival listed in Table 1^[^58^]^. Nonetheless, advances in RT, particularly SBRT and stereotactic radiosurgery (SRS), have significantly improved treatment options by enhancing local control, increasing resection rates, and preserving organ function across a wide range of tumor types. In the GROINSS-V II study^[^54^]^, surgical interventions were found to offer better local control compared to radiation in early-stage cervical cancer. However, surgery was associated with an increased risk of postoperative complications, such as lymphocele and pelvic infections. For high-risk patients with positive surgical margins or lymphovascular invasion, adjuvant radiation was shown to enhance local control, but it also raised the likelihood of long-term GI damage. RT, particularly SRS, is effective in treating brain metastases^[^54^]^. SRS offers better early local control than surgical resection, with lower recurrence rates and fewer side effects, such as postprocedural edema, making it a superior alternative for those with a limited number of brain metastases, especially when surgery is considered too hazardous.

Research into head and neck cancer has indicated that combining surgery and radiation can be effective. Studies comparing transoral surgery (TOS) with reduced-dose postoperative radiation to definitive RT for oropharyngeal cancer found that both procedures yielded comparable oncologic outcomes. However, the surgical cohort demonstrated better functional results, particularly in swallowing, with fewer occurrences of dysphagia^[^55^]^. Furthermore, TORS combined with reduced-dose radiation resulted in superior functional outcomes, such as enhanced swallowing function and QOL, compared to definitive RT alone, while preserving similar survival rates. These findings highlight the potential for combining surgery with reduced-dose radiation to improve oncologic efficiency while preserving function, particularly in malignancies where organ function is crucial for patient QOL^[^55^]^.

The effectiveness of surgery compared to other forms of radiation remains a key concern in the treatment of numerous cancers. A retrospective study of primary squamous cell carcinoma (PSCC) of the parotid gland found that surgical resection combined with adjuvant RT resulted in improved outcomes^[^55^]^. Patients who underwent parotidectomy had median survival duration of 24.3 months, with complete remission in eight of twelve cases. Non-surgical patients had a worse prognosis, with increased recurrence and mortality rates, emphasizing the significance of surgery in improving survival outcomes^[^55^]^. The study suggests that the best results are achieved when surgery is combined with RT. These findings indicate that, while SABT provides a non-invasive alternative to surgery, further research is needed to determine its usefulness in routine therapy.

A further examination of locally advanced recurrent nasopharyngeal cancer found that endoscopic surgery had slightly better outcomes than IMRT, with a 5-year OS of 68.4% for surgery and 65.4% for IMRT. Additionally, surgery was linked with fewer severe effects, such as temporal lobe necrosis, compared to IMRT^[^56^]^. In another cohort study on esthesioneuroblastoma, combining surgery with RT demonstrated high survival and recurrence-free results, emphasizing the aggressive nature of the tumor and the necessity of multimodal treatment in resectable cases^[^59^]^. A population-based retrospective analysis of biliary tract tumors underscored the significance of surgical intervention, particularly when paired with adjuvant chemotherapy (capecitabine), as the most effective method for enhancing survival in patients with resectable gallbladder and cholangiocarcinoma^[^60^]^.

In the case of oropharyngeal cancer, surgery combined with radiation resulted in higher survival rates (73.3%) compared to chemoradiotherapy alone (53.6%), demonstrating that surgery remains a cornerstone of treatment in later stages^[^57^]^. A retrospective cohort study comparing surgery and SBRT in early-stage NSCLC found that surgery improved survival rates, with a median overall survival rate of 48.1–64.6%, compared to 30.4% for SBRT. Even though SBRT is less invasive, surgery is preferred for operable patients due to the extended survival benefits^[^61^]^.

There are limitations in thoroughly evaluating functional outcomes associated with each treatment modality due to limited sample sizes, varied patient demographics, and inadequate long-term follow-up in the current research. More rigorous studies are necessary to enhance clinical decision-making. Despite its demonstrated efficacy in many tumor forms, RT has been repeatedly linked to more complications than surgery, particularly in terms of GI, hematologic, and long-term functional difficulties like dysphagia and xerostomia. Surgery, although effective for local management and often enhancing overall survival, presents its own set of risks, including an elevated incidence of postoperative complications, such as facial nerve palsy in parotidectomy patients.

### Future directions

One of the most advanced treatment approaches in radiation oncology is SBRT. SBRT delivers high-dose radiation to tumor cells (very efficiently and accurately), without any adverse effect on adjacent healthy cells. This therapy is specifically prescribed in cases that are medically ineligible for any surgical intervention. Not to mention the use of artificial intelligence in RT, which can access imaging data (like CT scan or MRI) to augment the therapeutic approach, ensures accurate radiation at tumor sites whilst mitigating the adverse effects (Table 1).

Robotic-assisted radical prostatectomy is certainly more successful in terms of decreased cancer recurrence and improved tumor excision rates as compared to the laparoscopy approach. Moreover, patients recover with similar functional outcomes in both approaches^[^62^]^. An in-depth evaluation is being carried out to implement robotic surgery in colorectal and gynecological cancers, which has the potential for effective management and enhanced patient outcome. Nonetheless, fluorescence-guided surgery (special lighting to highlight tumor margin) facilitates the procedure to ascertain the tumor precisely, thereby leading to better excision and enhanced outcome.

Ongoing research is being carried out which focuses on treatment plans based on unique characteristics of each tumor. The TAPUR study is exploring the success rate of the targeted treatment plan which is tailored to the genetic variation of different categories of cancer^[^63^]^. Another clinical trial PACIFIC-2 aimed at studying the efficacy of Durvalumab administered with chemoradiotherapy for stage 3 NSCLC. These studies aim to discover new strategies that can be implemented for better treatment and enhanced patient outcome^[^64^]^.

A combination of RT and surgery has exhibited better patient outcomes. Moreover, rectal cancer when treated by a neoadjuvant RT approach has proven to be beneficial for patients^[^64^]^. Innovations in treatment strategies accompanied by tailored medication and immune system-based therapies can lead to better patient outcomes and increased survival rates. However, there is a need to conduct careful investigation of the new therapeutic approaches to address the proper management of the disease.

## Conclusion

Surgical resection remains the cornerstone of treatment for resectable cancers, providing high rates of local control and survival. Recent advancements in RT, particularly SBRT and IMRT, have expanded its role as both an alternative and complementary modality. Although surgical intervention enables definitive tumor removal, RT offers significant advantages, such as organ preservation and enhanced clinical outcomes when integrated as neoadjuvant or adjuvant therapy. It is imperative for clinical practice to adopt a patient-centered approach, tailoring treatment strategies based on individual tumor characteristics and the associated risks of each modality. Future research should prioritize RCTs comparing contemporary RT techniques with surgical outcomes, assessing long-term complications such as secondary malignancies, and exploring multimodal therapeutic regimens. Such efforts will refine criteria for personalized treatment selection, optimizing patient care in the management of resectable malignancies.

## Data Availability

Available upon reasonable request.

## References

[1] Sankaranarayanan R. Screening for cancer in low- and middle-income countries. Ann Glob Health 2014;80:412. 25512156 10.1016/j.aogh.2014.09.014

[2] Wardle J Robb K Vernon S. Screening for prevention and early diagnosis of cancer. Am Psycholog 2015;70:119–33. 10.1037/a0037357 25730719

[3] Siegel RL Giaquinto AN Jemal A. Cancer statistics, 2024. CA Cancer J Clin 2024;74:12–49. 38230766 10.3322/caac.21820

[4] Diwanji TP Mohindra P Vyfhuis M. Advances in radiotherapy techniques and delivery for non-small cell lung cancer: benefits of intensity-modulated radiation therapy, proton therapy, and stereotactic body radiation therapy. Transl Lung Cancer Res 2017;6:131–47. 28529896 10.21037/tlcr.2017.04.04 PMC5420540

[5] Williamson CW Liu HC Mayadev J. Advances in external beam radiation therapy and brachytherapy for cervical cancer. Clin Oncol 2021;33:567–78. 10.1016/j.clon.2021.06.012 34266728

[6] Rocha PHP Reali RM Decnop M. Adverse radiation therapy effects in the treatment of head and neck tumors. RadioGraphics 2022;42:806–21. 35302867 10.1148/rg.210150

[7] Gómez Ruiz M Martín Parra I Palazuelos CM. Robotic-assisted laparoscopic transanal total mesorectal excision for rectal cancer. Dis Colon & Rectum 2015;58:145–53. 10.1097/DCR.0000000000000265 25489707

[8] Fico V Altieri G Di Grezia M. Surgical complications of oncological treatments: a narrative review. World J Gastrointest Surg 2023;15:1056–67. 37405101 10.4240/wjgs.v15.i6.1056 PMC10315125

[9] Brucker SY Ulrich UA. Surgical treatment of early-stage cervical cancer. Oncol Res Treat 2016;39:508–14. 27614875 10.1159/000448794

[10] Wallis CJD Saskin R Choo R. Surgery versus radiotherapy for clinically-localized prostate cancer: a systematic review and meta-analysis. Eur Urol 2016;70:21–30. 26700655 10.1016/j.eururo.2015.11.010

[11] Klimo P Thompson CJ Kestle JRW. A meta-analysis of surgery versus conventional radiotherapy for the treatment of metastatic spinal epidural disease. Neuro Oncol 2005;7:64–76. 15701283 10.1215/S1152851704000262 PMC1871618

[12] Baskar R Lee KA Yeo R. Cancer and radiation therapy: current advances and future directions. Int J Med Sci 2012;9:193–99. 22408567 10.7150/ijms.3635 PMC3298009

[13] Moding EJ Kastan MB Kirsch DG. Strategies for optimizing the response of cancer and normal tissues to radiation. Nat Rev Drug Discovery 2013;12:526–42. 23812271 10.1038/nrd4003 PMC3906736

[14] Keisuke SASAI. My 42-year experience in radiation oncology. Juntendo Med J 2022;68:332–38. 10.14789/jmj.JMJ22-0025-R PMC11250016 39021424

[15] Jaffray DA Das S Jacobs PM. How advances in imaging will affect precision radiation oncology. Int J Radiat Oncol*Biol*Phys 2018;101:292–98. 10.1016/j.ijrobp.2018.01.047 29726358

[16] Falkson CB Vella ET Yu E. Guideline for radiotherapy with curative intent in patients with early-stage medically inoperable non-small-cell lung cancer. Curr Oncol 2017;24:44–49. 10.3747/co.24.3358 PMC5330637 28270731

[17] Moore S Leung B Wu J. Population-based analysis of curative therapies in stage II non-small cell lung cancer: the role of radiotherapy in medically inoperable patients. Radiat Oncol 2020;15:23. 32000829 10.1186/s13014-020-1466-y PMC6993511

[18] Sathiyapalan A Baloush Z Ellis PM. Update on the management of stage III NSCLC: navigating a complex and heterogeneous stage of disease. Curr Oncol 2023;30:9514–29. 37999109 10.3390/curroncol30110689 PMC10670056

[19] Ohri N Haffty BG. The evolution of adjuvant radiation therapy for early-stage and locally advanced breast cancer. Breast J 2019;26:59–64. 31854499 10.1111/tbj.13715

[20] Cooper JS Zhang Q Pajak TF. Long-term follow-up of the RTOG 9501/intergroup phase III trial: postoperative concurrent radiation therapy and chemotherapy in high-risk squamous cell carcinoma of the head and neck. Int J Radiat Oncol Biol Phys 2012;84:1198–205. 22749632 10.1016/j.ijrobp.2012.05.008 PMC3465463

[21] Sanábria Á Lauda A Kowalski LP. Organ preservation with chemoradiation in advanced laryngeal cancer: the problem of generalizing results from randomized controlled trials. Auris Nasus Larynx 2017;44:18–25. 27397024 10.1016/j.anl.2016.06.005

[22] Falick T Blumenfeld P Skripai A. The effect of radiotherapy on the course of disease in prostatic cancer with high metastatic burden. J Clin Oncol 2024;42:112–112.

[23] Lazarev S Gupta V Ghiassi-Nejad Z. Premature discontinuation of curative radiation therapy: insights from head and neck irradiation. Adv Radiat Oncol 2018;3:62–69. 29556582 10.1016/j.adro.2017.10.006 PMC5856974

[24] Jochems A El-Naqa I Kessler M. A prediction model for early death in non-small cell lung cancer patients following curative-intent chemoradiotherapy. Acta Oncologica 2017;57:226–30. 29034756 10.1080/0284186X.2017.1385842 PMC6108087

[25] Fekete Z Fekete A Kacsó G. Treatment classification by intent in oncology—the need for meaningful definitions: curative, palliative and potentially life-prolonging. J Pers Med 2024;14:932–932. 39338185 10.3390/jpm14090932 PMC11433215

[26] Chouhan R Patel R Shakib K. Surgical excision of non-melanoma skin cancer: no end in site? British J of Oral and Maxillofacial Surg 2021;59:1264–69. 10.1016/j.bjoms.2021.05.018 34275678

[27] Muto Y Okuma Y. Therapeutic options in thymomas and thymic carcinomas. Expert Rev Anticancer Ther 2022;22:401–13. 35266421 10.1080/14737140.2022.2052278

[28] Aggarwal P Knabel P Fleischer AB. United States burden of melanoma and non-melanoma skin cancer from 1990 to 2019. J Am Acad Dermatol 2021;85:388–95. 33852922 10.1016/j.jaad.2021.03.109

[29] Agarwal S Pappas L Neumayer L. Effect of breast conservation therapy vs mastectomy on disease-specific survival for early-stage breast cancer. JAMA Surg 2014;149:267. 24429935 10.1001/jamasurg.2013.3049

[30] van Maaren MC de Munck L de Bock GH. 10 year survival after breast-conserving surgery plus radiotherapy compared with mastectomy in early breast cancer in the Netherlands: a population-based study. Lancet Oncol 2016;17:1158–70. 27344114 10.1016/S1470-2045(16)30067-5

[31] Plante M Kwon JS Ferguson S. Simple versus radical hysterectomy in women with low-risk cervical cancer. N Engl J Med 2024;390:819–29. 38416430 10.1056/NEJMoa2308900

[32] Kees J Broekman MLD De Vleeschouwer S. Safe surgery for glioblastoma: recent advances and modern challenges. Neuro-Oncol Pract 2022;9:364–79. 10.1093/nop/npac019 PMC9476986 36127890

[33] Huber T Tripke V Baumgart J. Computer-assisted intraoperative 3D-navigation for liver surgery: a prospective randomized-controlled pilot study. Ann Transl Med 2023;11:346–346. 37675318 10.21037/atm-22-5489 PMC10477660

[34] Ould-Slimane M Thong P Perez A. The role of Intraoperative 3D navigation for pelvic bone tumor resection. Ortho Traumatol 2016;102:807–11. 10.1016/j.otsr.2016.03.019 27318805

[35] Gradishar WJ Moran MS Abraham J. Breast cancer, version 3.2024, NCCN clinical practice guidelines in oncology. J Natl Compr Cancer Network 2024;22:331–57. 10.6004/jnccn.2024.0035 39019058

[36] Zane KE Nagib PB Jalil S. Emerging curative-intent minimally-invasive therapies for hepatocellular carcinoma. World J Hepatol 2022;14:885–95. 35721283 10.4254/wjh.v14.i5.885 PMC9157708

[37] Lee G Strickland MR Wo JY. Role of preoperative radiation therapy for resectable gastric cancer. J Gastrointest Cancer 2024;55:584–98. 38353901 10.1007/s12029-023-00985-6

[38] Liu B Wang Z Zhao H. The value of radiotherapy in patients with resectable stage IIIA non–small-cell lung cancer in the Era of individualized treatment: a population-based analysis. Clin Lung Cancer 2023;24:18–28. 36446703 10.1016/j.cllc.2022.09.011

[39] Brozzetti S D’Alterio C Bini S. Surgical resection is superior to TACE in the treatment of HCC in a well selected cohort of BCLC-B elderly patients—a retrospective observational study. Cancers (Basel) 2022;14:4422–4422. 36139581 10.3390/cancers14184422 PMC9496726

[40] Tamburrino D. Selection criteria in resectable pancreatic cancer: a biological and morphological approach. World J Gastroenterol 2014;20:11210. 25170205 10.3748/wjg.v20.i32.11210 PMC4145759

[41] Stefanidis K Konstantellou E Yusuf G. The evolving landscape of lung cancer surgical resection: an update for radiologists with focus on key chest CT findings. Am J Roentgenol 2022;218:52–65. 34406062 10.2214/AJR.21.26408

[42] Pedro C Mira B Silva P. Surgery vs. primary radiotherapy in early-stage oropharyngeal cancer. Clin Transl Radiat Oncol 2018;9:18–22. 29594246 10.1016/j.ctro.2017.12.002 PMC5862671

[43] Gong W Zhao H Liu S. Outcomes of radiation therapy for resectable M0 gastric cancer. Oncotarget 2017;9:1726–34. 29416726 10.18632/oncotarget.22574 PMC5788594

[44] Majeed H Gupta V. Adverse effects of radiation therapy. PubMed. Accessed August 14, 2023. https://www.ncbi.nlm.nih.gov/books/NBK563259/33085406

[45] Pak H Maghsoudi LH Soltanian A. Surgical complications in colorectal cancer patients. Ann Med Surg 2020;55:13–18. 10.1016/j.amsu.2020.04.024 PMC7229272 32435475

[46] Zhan TC Zhang DK Gu J. Surgical complications after different therapeutic approaches for locally advanced rectal cancer. World J Gastrointest Oncol 2019;11:393–403. 31139309 10.4251/wjgo.v11.i5.393 PMC6522767

[47] Li R Xu Y Zhao J. Comparison of radiotherapy versus surgical resection following neoadjuvant chemoimmunotherapy in potentially resectable stage III non-small-cell lung cancer: a propensity score matching analysis. Lung Cancer 2024;194:107884. 38991281 10.1016/j.lungcan.2024.107884

[48] Thunshelle CP Kouzy R Jaoude JA. Patient-reported outcomes in prospective cohort of patients with borderline resectable or locally advanced pancreatic cancer undergoing radiotherapy. Int J Radiat Oncol*Biol*Phys 2020;108:e628.

[49] Achim V Bolognone RK Palmer AD. Long-term functional and quality-of-life outcomes after transoral robotic surgery in patients with oropharyngeal cancer. JAMA Otolaryngol–Head & Neck Surg 2017;144:18-27. 10.1001/jamaoto.2017.1790 PMC5833591 29075740

[50] Douglas C Hewitt L Yabe TE. Quality of life impacts following surgery for advanced head and neck cancer. World J Oncol 2023;14:150–57. 37188036 10.14740/wjon1541 PMC10181422

[51] Sheng WG Assogba E Billa O. Does baseline quality of life predict the occurrence of complications in resectable esophageal cancer? Surg Oncol 2022;40:101707. 35030410 10.1016/j.suronc.2021.101707

[52] Dohopolski MJ Diao K Hutcheson KA. Long-term patient-reported outcomes in a population-based cohort following radiotherapy vs surgery for oropharyngeal cancer. JAMA Otolaryngol–Head & Neck Surg 2023;149:697. 37382943 10.1001/jamaoto.2023.1323 PMC10311423

[53] Oonk M Slomovitz BM Baldwin P. Radiotherapy versus inguinofemoral lymphadenectomy as treatment for vulvar cancer patients with micrometastases in the sentinel node: results of GROINSS-V II. J Clin Oncol 2021;39:3623–32. 34432481 10.1200/JCO.21.00006 PMC8577685

[54] Churilla TM Chowdhury IH Handorf E. Comparison of local control of brain metastases with stereotactic radiosurgery vs surgical resection. JAMA Oncol 2019;5:243. 30419088 10.1001/jamaoncol.2018.4610 PMC6439566

[55] Brennan P Flamand Y Weinstein GS. Phase II randomized trial of transoral surgery and low-dose intensity modulated radiation therapy in resectable p16+ locally advanced oropharynx cancer: an ECOG-ACRIN cancer research group trial (E3311). J Clin Oncol 2022;40:138–49. 34699271 10.1200/JCO.21.01752 PMC8718241

[56] Liu YP Wen YH Tang J. Endoscopic surgery compared with intensity-modulated radiotherapy in resectable locally recurrent nasopharyngeal carcinoma: a multicentre, open-label, randomised, controlled, phase 3 trial. Lancet Oncol 2021;22:381–90. 33600761 10.1016/S1470-2045(20)30673-2

[57] Caldeira PC Bonardi MJF Pantuzzo ERM. Advanced carcinoma of the oropharynx: survival analysis comparing two treatment modalities. Braz Oral Res 2020;34:e032. 32267289 10.1590/1807-3107bor-2020.vol34.0032

[58] Papaconstantinou D Tsilimigras DI Moris D. Synchronous resection of esophageal cancer and other organ malignancies: a systematic review. World J Gastroenterol 2019;25:3438–49. 31341367 10.3748/wjg.v25.i26.3438 PMC6639548

[59] Alami Z Farhane FZ Bouziane A. Management of esthesioneuroblastoma: a retrospective study of 6 cases and literature review. Case Rep Oncol 2022;15:176–86. 35431860 10.1159/000521736 PMC8958627

[60] Beaulieu C Lui A Yusuf D. A population-based retrospective study of biliary tract cancers in Alberta, Canada. Curr Oncol 2021;28:417–27. 33450805 10.3390/curroncol28010044 PMC7903272

[61] Chi A Fang W Sun Y. Comparison of long-term survival of patients with early-stage non–small cell lung cancer after surgery vs stereotactic body radiotherapy. JAMA Network Open 2019;2:e1915724. 31747032 10.1001/jamanetworkopen.2019.15724 PMC6902813

[62] Novara G Ficarra V Mocellin S. Systematic review and meta-analysis of studies reporting oncologic outcome after robot-assisted radical prostatectomy. Eur Urol 2012;62:382–404. 22749851 10.1016/j.eururo.2012.05.047

[63] Adusumilli P. PL03.05 cell therapy for solid tumors. J Thoracic Oncol 2021;16:S65–S66.

[64] Ngan SY Burmeister B Fisher RJ. Randomized trial of short-course radiotherapy versus long-course chemoradiation comparing rates of local recurrence in patients with T3 rectal cancer: trans-tasman radiation oncology group trial 01.04. J Clin Oncol 2012;30:3827–33. 23008301 10.1200/JCO.2012.42.9597

